# Feasibility Testing of a Health Literacy Intervention With Adolescents and Young Adults in South Africa: The LifeLab Soweto Programme

**DOI:** 10.1111/hex.70121

**Published:** 2024-12-11

**Authors:** Lisa J. Ware, Delisile Kubheka, Thato Mdladlamba, Khuthala Mabetha, Mark Hanson, Keith M. Godfrey, Kathryn Woods‐Townsend, Shane Norris

**Affiliations:** ^1^ Wits Health Hubb, Wits Health Consortium Johannesburg South Africa; ^2^ SA MRC‐Wits Developmental Pathways for Health Research Unit, Department of Paediatrics, Faculty of Health Sciences, School of Clinical Medicine University of the Witwatersrand Johannesburg South Africa; ^3^ DSI‐NRF Centre of Excellence in Human Development University of the Witwatersrand Johannesburg South Africa; ^4^ NIHR Southampton Biomedical Research Centre University of Southampton and University Hospital Southampton NHS Foundation Trust Southampton UK; ^5^ MRC Lifecourse Epidemiology Centre University of Southampton Southampton UK; ^6^ School of Healthcare Enterprise and Innovation University of Southampton Southampton UK; ^7^ School of Health and Human Development University of Southampton Southampton UK

**Keywords:** adolescents, health literacy, South Africa, young adults

## Abstract

**Introduction:**

Low health literacy levels during adolescence and young adulthood (AYA) may impact acute healthcare access and longer‐term health outcomes. Previous research in South African AYA suggests that health literacy levels are typically suboptimal but few interventions exist. This study aimed to test the acceptability and feasibility of a co‐created, interactive health literacy intervention (LifeLab‐Soweto) with AYA in Soweto, South Africa.

**Methods:**

Participants (18–24 years, *n* = 107) were recruited (September–October 2022) from a youth development centre database by telephone and through snowball sampling. AYA involved in the co‐creation process were excluded. Pre‐intervention data on participant age, gender identity and ability to correctly identify a normal blood pressure (BP) reading were captured via survey. Post‐intervention, participants repeated the BP question and completed a satisfaction survey. Additionally, *n* = 31 AYA agreed to an in‐depth interview about their LifeLab‐Soweto experience. Interview transcripts were analysed using inductive thematic analysis.

**Results:**

Participants (mean age 21 ± 2.4 years; 59% female, 39% male, 2% nonbinary) generally viewed LifeLab‐Soweto as well‐designed, relevant, simple to follow, fun, useful, and interesting, with most reporting an increased understanding of health and that they would use this new knowledge. Comparing pre‐ and post‐intervention BP question accuracy, males showed the greatest improvement in scores. Interviews showed that, while LifeLab‐Soweto was not what AYA were expecting, gains in health knowledge led AYA to consider changes in health behaviours including accessing health services.

**Conclusion:**

Life‐Soweto presents an acceptable, feasible and relevant health literacy intervention for South African youth with potential to improve health literacy and health behaviours.

**Patient and Public Involvement:**

To ensure the health literacy intervention was contextually relevant, age appropriate, and gender inclusive, a group of 40 adolescents (aged 18–24 years, male, female and non‐binary) were recruited from Soweto to firstly identify the health topics that were most pressing in their daily lives. This youth advisory group identified stress as a major challenge impacting physical and mental health, health behaviour and daily functioning. Together with the youth group, researchers from South Africa and the UK worked to co‐develop the health literacy intervention that delivers self‐directed exploration and learning of how stress impacts health, behaviour and well‐being. This manuscript describes how this cocreated intervention was received by a broader range of South African youth who were not involved in the cocreation process.

## 1 Introduction

Health literacy is a critical determinant of health that influences how individuals and communities use and contribute to health systems, take personal responsibility for health, and advocate to address the broader social and political drivers of health inequities and access to care [1]. Personal health literacy is defined as the extent to which individuals have the ability to find, understand, and use information and services to inform their health‐related behaviours and decisions for themselves and others [2, 3]. Recent guidelines further include a definition for organisational health literacy as ‘the degree to which organisations equitably enable individuals to find, understand, and use information and services to inform health‐related decisions and actions for themselves and others’ [3], thereby placing responsibility on organisations to support gains in personal health literacy. Meeting the health literacy needs, particularly of the most disadvantaged and marginalised communities, is likely to accelerate progress in reducing inequalities in health and beyond [4]. Low health literacy can have critical health implications including more primary care visits, more hospital admissions and longer hospital stays, especially for those dealing with complex chronic conditions [5].

Health literacy in adolescents and young adults (AYA) is important as this is a time in life when health behaviours are cemented, and where low health literacy may already increase risk for poor cardiovascular health [6]. Although health literacy research is generally increasing globally [7], studies in adolescents remain scarce, especially in African adolescents [8]. The studies that do exist suggest that low health literacy is commonly observed in African adolescents and associated with poor health outcomes [8].

To evaluate health literacy specifically in South Africa, the Health literacy test for limited literacy populations (HELT‐LL) was developed [9]. Using this test, prior research has shown that only one in five South African adults (18%) in both rural and urban areas had adequate health literacy [9, 10]. Despite these low health literacy levels and the requirement to increase health literacy beginning in youth, a significant gap exists for effective health literacy interventions for adolescents in the South African context.

LifeLab is a health literacy intervention developed in the UK for school‐based implementation with adolescents and has been shown to improve health and scientific literacy [11]. In the original setting, this intervention includes a teaching guide [12] and professional training for teachers to facilitate a 2–3‐week health literacy training programme with adolescent students, drawing upon principles of education, psychology and public health [13]. The key programmatic theme is ‘Me, my health and my children's health’ built upon the ‘developmental origins of health and disease’ (DOHaD) concept [14]. The LifeLab programme concludes with a ‘hands‐on’ practical health science day conducted in the LifeLab centre, hosted within a local university teaching hospital. Topic areas include health behaviours (e.g., sleep, physical activity, substance misuse and food choices), mental health, and social and environmental factors that influence health, and the use of a simple LifeLab video to show using cartoons how health is transmitted from one generation to the next (based on the concept of epigenetics). The programme has successfully been implemented in schools with disadvantaged adolescents in Dublin, Ireland [15] following local co‐development activities [16] and similar implementation is currently underway with schools in Sydney, Australia.

However, within the South African context, implementing such programmes within the school curriculum can be challenging as teachers struggle to deliver basic sexual and reproductive health education [17]. Additionally, the health concerns and priorities of UK adolescents and South African adolescents are likely to be quite different and for health literacy interventions to be successful, they must speak to the needs and values of the population of interest. Therefore, we conducted a series of focus groups and workshops with urban adolescents and young adults (AYA) in Soweto, South Africa to cocreate a contextually relevant health literacy intervention in collaboration with and utilising the materials and learnings from the LifeLab UK team [18]. The aim of this study was to test the feasibility of this co‐created LifeLab‐Soweto intervention with other AYA not involved in its co‐creation, to understand their engagement with and perceptions of the intervention, as well as test the potential impact on knowledge of blood pressure as a simple measure of health.

## 2 Methods

### 2.1 Participants, Setting and Recruitment

The study took place in South Africa, at a community youth development centre located centrally in Soweto, a historically disadvantaged urban area on the outskirts of Johannesburg with an estimated population of almost 1.7 million people [19]. The centre opened in 2021 through a Development Bank of Southern Africa (DBSA) social development infrastructure initiative in partnership with the City of Johannesburg and was created to provide a safe space for youth to play, learn and develop skills to enhance employability. AYA aged 18–24 years were contacted by telephone from registers held at the youth development centre and given at least 24 h to consider if they would like to take part. All youth who register at the centre give their consent to be contacted for opportunities to join programmes or sign up for activities including studies conducted at the centre. Additional AYA were recruited through snowball sampling. Those AYA who had been involved in the co‐creation of LifeLab‐Soweto were excluded from taking part in this feasibility study.

### 2.2 Ethical Statement

Approval to conduct the study was granted by the Human Research Ethics Committee (Medical) based at the University of the Witwatersrand [M220438]. All participants gave written informed consent to participate and for the group discussions to be audio recorded before data collection.

### 2.3 LifeLab‐Soweto Intervention

Participants were invited to attend an in‐person session between September and October 2022 to experience and explore the LifeLab‐Soweto health literacy programme through guided experiential learning. The prior co‐creation workshops with AYA locally [18] resulted in an intervention focused on understanding the impact of acute and chronic stress (the topic identified by the AYA co‐creation group as most salient to daily health) on both physical and mental health. Sections in the booklet included guided activities to examine the impact of stress on exercise, blood pressure, memory, sleep, brain function, nutrition, and health behaviours such as smoking. Exploratory ‘activity stations’ were set up in a large room for AYA to assess how these various aspects of their health were impacted by stress. After a brief introduction from the research team, the LifeLab‐Soweto booklet was given to each AYA to guide the learners on a journey around the activity stations and prompt AYA to record their own results next to the results of two fictional characters in the booklet: a fictional character of the same age as the AYA (Sifiso) and his aunt. Examples of the activity stations included a set of scales and a stadiometer with instructions on how to measure height and weight, alongside a chart to identify Body Mass Index (BMI) and determine BMI category. This aligned with the booklet section detailing how stress had impacted the physical activity and dietary behaviours of the fictional characters and how this had impacted their body weight and BMI. Other examples included activities to explore the impact of stress on concentration and coordination (Buzz wire task), and on problem solving, planning, and working memory (Tower of Hanoi game) [20, 21], with examples in the booklets of how stress had impacted these processes in the fictional characters.

**Figure 1:**
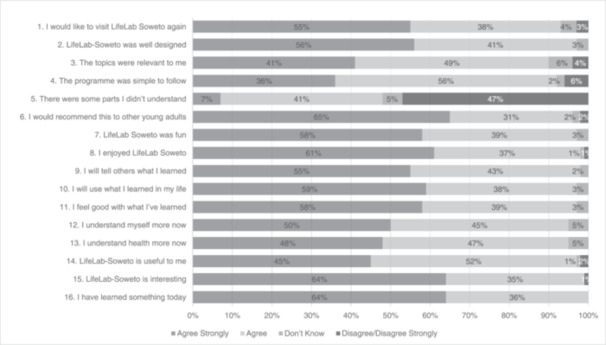
Opinions of the LifeLab‐Soweto health literacy intervention (*n* = 107).

The exercises and storylines were previously co‐created with AYA in Soweto to show the impact of stress on physical and mental health, performance, and behaviour and to avoid stigmatisation through reviewing stories and results of persons designed to be ‘like them’ (see Supporting Information S2: Figure 1, for an extract of the storylines). This approach has been employed across multiple LifeLab sites [22] and allows AYA to engage with learning in a de‐personalised way, while internalising the discussions to reflect on their own lives. Groups of 10 AYA at a time were given access to the LifeLab‐Soweto installation. While AYA explored the activities individually or in pairs, each recorded their results in their own LifeLab‐Soweto booklet which was then given to them to keep for reference and reflection. Research team members were available for questions throughout the sessions or for support should any AYA exhibit distress. Furthermore, contact details for local youth‐friendly physical and mental health service providers were included in the back cover of the booklet, alongside guidance on how to design their own plan to support improving health and health behaviours.

### 2.4 Data Collection

Before the intervention, participants completed a brief online survey using the REDCap electronic data capture tool [23] to record their age, gender identity and to determine if they could correctly identify a normal blood pressure (BP in mmHg) reading for their age group from three available options (150/100, 90/160 or 120/80), with the last option (120/80) being the correct answer. Immediately following the LifeLab‐Soweto intervention, participants were asked the same question to determine if BP knowledge had changed and completed an online survey about their experience of the LifeLab‐Soweto programme. The AYA participants were then asked if they would be willing to undertake a further in‐depth interview. For those that agreed, the in‐depth interview was conducted using a semi‐structured interview topic guide (Box 1) by a trained multilingual qualitative interviewer in the language in which participants were most comfortable (predominantly English, isiZulu, Sesotho, Setswana).

Box 1 **Semi‐Structured Interview Topic Guide**

1 How did you find out about LifeLab‐Soweto? What did you think when you heard about it? What did you expect the programme would be like?

What were your first thoughts when you arrived at the centre and met the programme team?

How did you experience the LifeLab‐Soweto programme? What did you like? What do you think was most useful for you?

How much did you know about these health topics before you attended the programme? How much have you learned from the programme?

What knowledge did you pick up? What skills have you learned? How will you use the knowledge or skills you've gained?

What didn't you like? What do you think could be better? How would you change this?

How easy or difficult did you find it to understand the programme content?

How approachable were the team delivering the programme? How knowledgeable did they seem to you? How comfortable did you feel asking them questions? Could they answer all your questions?

What do you think is most important for young people to know about health? What do others in the community need to know about their health? What do you think is the reason why people in your community sometimes struggle to understand these things about health?

How did you think your general health was before today? What factors contributed to this?

How do you think your general health is now after doing the programme?

What would you change about your current health? How would you achieve this?

Do you have anything else you would like to tell us about LifeLab‐Soweto?

### 2.5 Data Analysis

Pre‐ and post‐survey responses were compared for all participants combined, and separately by gender identity, with differences tested using Chi‐square tests for categorical data. Interviews were transcribed verbatim and translated where necessary by individuals fluent in local languages. All translations were cross‐checked by a second qualitative researcher fluent in local languages, and the transcripts analysed using thematic inductive analysis, following the guidance of Braun and Clarke [24]. Initial codes were developed by three research team members (D.K., T.M. and L.J.W.) after reading and rereading a subset of transcripts. The coders then developed a coding framework (Supporting Information S1: Table 1) which was reviewed by the research team to extract and describe the identified themes. Two coders (D.K., L.J.W.) then coded all transcripts and the level of agreement between the two coders in assigning the same codes to the same sections of transcripts was calculated to be 93%.

## 3 Results

Overall, 107 young people took part in the study. The mean age of the group was 21 ± 2.4 years; 59% identified as female, 39% identified as male and 2% as non‐binary (Table 1). Before the intervention, females were significantly more likely to correctly answer the blood pressure question. Following the intervention, the number of correct responses increased for both males and females, almost doubling in the male group, so that there was no longer a significant difference by gender in the number of correct responses.

**Table 1:** Change in blood pressure (BP) knowledge, according to participant characteristic.

	All (*n* = 107)	Male (*n* = 42)	Female (*n* = 63)	Nonbinary (*n* = 2)	Gender difference *p*‐value
Mean age	21.1 ± 2.4	21.3 ± 2.3	21.0 ± 2.5	20.5 ± 0.7	0.38
Pre‐test BP question correct, % (*n*)	41.1 (44)	23.8 (10)	50.8 (32)	100 (2)	**0.005**
Posttest BP question correct, % (*n*)	54.2 (58)	45.2 (19)	58.7 (37)	100 (2)	0.168
I would like to be interviewed about my experience with LifeLab‐Soweto, % (*n*)	64.5 (69)	81.0 (34)	52.4 (33)	100 (2)	**0.006**

Opinion about the LifeLab‐Soweto intervention was largely very positive (Figure 1), with many participants agreeing that the intervention was well‐designed, relevant, simple to follow, fun, useful, enjoyable, and interesting. Most participants also agreed that they had learned something, increased their understanding of themselves and their health, and that they felt good about this new knowledge and would use what they had learned as well as tell others and recommend the programme to others. However, almost half of the participants (48%) highlighted that there were some parts that they didn't understand.

### 3.1 In‐Depth Interviews

In total, 31 interviews were conducted (17 males, 13 females, 1 nonbinary) lasting an average of 29 ± 7 min. From the analysis of the interview transcripts, four themes were identified: (1) expectations of the LifeLab‐Soweto intervention; (2) experiences of the LifeLab‐Soweto intervention; (3) Learnings from the LifeLab‐Soweto intervention; and (4) Impact of the LifeLab‐Soweto intervention.

### 3.2 Theme 1. The LifeLab‐Soweto Intervention was not What Youth Expected

Participants described what they had anticipated happening during the LifeLab‐Soweto intervention before attending the session. The focus on health and the location at the youth development centre appeared confusing at first as several participants expected a more formal health education session led by healthcare professionals.

I didn't think much of health because the first thing I saw were games, *[…]* it's not what I expected, I thought maybe I would find nurses.

(Male – 010)

…when I arrived here I, I thought that okay maybe it's just a health survey.

(Male – 011)

I thought it was some sort of discussion on how to keep healthy amongst us the youth.

(Male – 012)

### 3.3 Theme 2. Youth Found LifeLab‐Soweto Challenging but Enjoyable

Participants quickly identified elements of LifeLab‐Soweto that they liked or found challenging, and those that were familiar to them through prior interactions with the health service or through media channels. The elements of the programme cited as most challenging were those exploring the impact of stress on concentration and coordination (Buzz wire task), and on problem solving, planning, and working memory (Tower of Hanoi game). In the few instances where participants did not understand how to interact with the programme, they described getting support from the research team. Overall, the programme elicited positive experiences in line with the survey results.

…the wire buzzer *[…]* the first time I attempted it I almost won but I fumbled a bit then I started afresh… the tower of Hanoi beat me. I had three attempts until I could finish it the fourth time.

(Male – 003)

I knew about the height and the buzzer because I usually saw it on TV *[…]* and the blood pressure *[…]* I knew it from the clinic.

(Male – 003)

I didn't know what to do [during the LifeLab programme] so I asked him [LifeLab team member]. He did deliver the programme well.

(Female – 009)

The part that I liked the most is that it's educative, fun, [and] it teaches us about health.

(Male – 012)

### 3.4 Theme 3. Youth Learned About Stress and How It Impacts Health

Participants described learning both new health knowledge and building on prior knowledge to achieve a better understanding of health including learning about epigenetics and getting to know their own blood pressure, body size (i.e. height, weight, waist circumference, BMI), flexibility, and ability to focus or concentrate on tasks (for quotes see Supporting Information S1: Table 1). Participants also described how they liked learning new skills including how to take BP or height measures, and how to better manage stress and to improve both physical and mental health. How LifeLab‐Soweto built upon the health education that AYA received in school at life orientation classes was also mentioned, including how LifeLab‐Soweto went deeper into the impact of stress. While females reported learning more specifically about stress, including the types of stress, males more frequently reported learnings about their overall health.

I didn't know much about stress. *[…]* I didn't know about chronic and acute [stress]. I now know the difference.

(Female – 015)

I knew about stress, heart disease and blood pressure, but I just knew the basics *[…]* I didn't know more.

(Female – 013)

I learnt all about health, keeping our body healthy, exercising, and keeping a healthy diet.

(Male – 012)

### 3.5 Theme 4. LifeLab‐Soweto Triggered Intention to Change Health Behaviour

Several participants reported they intended to make health changes after experiencing the LifeLab‐Soweto programme. This included wanting to share their learning with others and voicing actionable plans to sleep better and exercise more, with intentions to improve their diet and review their smoking habits. Several participants said how LifeLab‐Soweto had altered their perspectives on health, including realising how their health habits could impact their future offspring. A desire to engage more with general health services was also reported following LifeLab‐Soweto exposure.

I've also decided to join yoga classes so I can stretch a bit.

(Male – 010)

I'm starting with yoga tomorrow.

(Female – 013)

…one other thing I will change is to seek counselling.

(Female – 017)

I'd like to go for regular check‐ups *[…]* maybe once in 3 months…

(Male – 011)

## 4 Discussion

This study aimed to test the feasibility of a co‐created health literacy intervention (LifeLab‐Soweto) with South African adolescents in a low‐income urban area. Our findings demonstrate that the intervention was acceptable, well understood and liked by most participants but with some parts that they didn't understand. Additionally, engagement with the LifeLab‐Soweto programme resulted in participants reporting an increased understanding of health within the context of the information provided by the intervention, and an increased intention to engage in physical activity and health screening.

Our findings also indicate that young South African men specifically may gain more knowledge from such interventions compared to their female counterparts. This may be due to young men accessing healthcare services less frequently than young women and viewing clinics as places for women [25]. The young women who took part in our study were significantly more familiar with accurate BP readings before the intervention, with less gain post‐intervention. In part, this may be due to women typically having higher levels of engagement with primary care for sexual and reproductive healthcare services with the South African National Contraception Clinical Guidelines specifying that BP should be assessed at each visit [26]. In fact, a random sample survey conducted in three large primary care clinics in our province showed thirteen times as many young women attended the clinic compared to young men (age 16–25 years) [27]. Amongst a sample of tertiary education students, this gender disparity was also observed with six times as many females than males (age 18–25 years) engaging with family planning healthcare services in the 6 months before the survey [28]. In this regard, our qualitative indication from several young men that they intended to access healthcare screening following the LifeLab‐Soweto intervention is promising. However, evidence suggests that primary care services for young people generally in South Africa require improvement [29] such that primary care may not provide the optimum location for improving youth health literacy.

Previous interventions with young men to improve health behaviours have shown limited success in the region. For example, an intervention to reduce risky sexual behaviours of young men showed little change in behavioural intentions despite co‐development of the intervention curriculum and the delivery of multiple sessions over a 4‐week period [30]. While there were some skills transfer activities, such as role‐playing communication scenarios or how to put on a condom, much of the intervention focus was on introducing new topics and facilitating small group discussions with facilitators recruited from the local area. The authors concluded that the limited intervention effect may have been due in part due to too much focus on information provision and not specifically including ways to overcome stigma. Through relating to the fictional characters embedded in the booklet, LifeLab‐Soweto offers a way for youth to have a depersonalised experience of the health literacy intervention, while being able to compare their own results for reference and reflection. Furthermore, these results are generated by themselves, recorded in their own booklet, and not shared with the group. These approaches are designed specifically to reduce the risk of stigmatisation and to foster engagement with the intervention. Furthermore, LifeLab‐Soweto is delivered in an interactive and highly engaging format, developed by experts in educational pedagogy, as described previously [13, 31]. The approach is designed to make health and scientific knowledge both contextually relevant and accessible for youth.

Despite evidence from multiple African countries of low youth health literacy levels that relate to worse health behaviours and outcomes, health literacy studies and interventions in young people in the region remain scarce [8, 32]. The interventions that have been tested in African AYA have largely focused on information provision. For example, a school‐based intervention in Nigeria challenged 10–18‐year olds to evaluate their negative attitudes towards people with mental illness through increasing mental health literacy using information provision and facilitated group discussion (one 3‐h session) [33]. Despite increasing knowledge, youth showed no intention to alter attitudes toward people with poor mental health. This school‐based intervention in Nigeria was modified from a previously successful programme in the UK. While LifeLab‐Soweto was also adapted from a successful UK‐based programme, a critical difference may be the involvement of the young people themselves in the creation of the Soweto programme, as previously reported [34].

Where more interactive health literacy interventions exist, these appear to support a greater increase in health knowledge. For example, an intervention delivered during a South African National Science exhibition to improve diabetes knowledge in junior and secondary school children included an interactive quiz and presentation, models to explore, a word search game, a practical demonstration of measuring body mass index (BMI) and blood pressure with an option to self‐measure, posters and (bilingual) leaflets [35]. Parents and teachers attended the session with children and significant increases in diabetes knowledge were observed for both younger and older children. However, intentions for changing behaviour were not assessed and the intervention design was driven by pharmacy students.

The successful LifeLab‐UK programme is founded on solid educational pedagogies and uses evidence‐based behaviour change techniques. It is co‐created with end users, delivered collaboratively with secondary school teachers, and embedded into multiple sessions within the school science curriculum including a session where learners visit the LifeLab‐UK laboratory for a hands‐on experiential learning day. Additionally, teachers are provided with a professional development programme to support LifeLab‐UK delivery. While there are examples of embedding health literacy programmes into schools in Africa, delivering these collaboratively with teachers and embedding these within curricula may be more challenging when resources are often scarcer. A programme in Ugandan schools to increase HIV health literacy and improve HIV‐related beliefs and prevention attitudes was delivered across multiple monthly sessions with adolescents, showing improvements on all measures, though this relied on peer‐mentoring to deliver the programme [36]. It remains to be seen if an interactive and co‐created health literacy intervention can be fully integrated into the school curriculum within Africa.

The results of this study should be considered taking into account several limitations. Firstly, this was a relatively modest sample of adolescents and conducted with youth from a defined geographical area, limiting the generalisability of the study. However, the in‐depth interviews, in addition to the survey data provided rich information about the feasibility of this approach in a low‐income African community and supports the need for further research to test the impact of LifeLab‐Soweto. Further limitations lie in not assessing the educational background or health literacy level of the adolescents before testing the intervention as this may have influenced how they experienced LifeLab‐Soweto. However, the aim was to evaluate the intervention with youth generally and not to select by educational level or baseline health literacy, this will be evaluated in further studies to determine if subsets of youth may need additional support with the intervention. Lastly, we did not check if the results that the youth recorded for themselves were accurate. We have shown in previous research that adults in Soweto are able to accurately measure several health indicators at home following instruction by a community health worker [10]. However, LifeLab‐Soweto was not designed to support self‐diagnosis, only learning and engagement with the topic and youth who were concerned about results could be referred for retesting to appropriate healthcare service providers. While future studies could evaluate AYA measurement accuracy, these should also assess the impact of the study team checking AYA recorded results on youth engagement as there may be a risk of reduced engagement due to fear that performance and results are being evaluated. Furthermore, the booklet is designed to support self‐guided learning so that delivery of the intervention requires minimal resource, an important consideration for our context. A strength of this study lies in testing the feasibility of LifeLab‐Soweto with youth who were not involved in the contextual adaptation and co‐creation of the programme. Furthermore, this is the first evaluation of the LifeLab programme within an African context and with delivery outside of the schooling system.

According to Bowen et al. feasibility testing is warranted when an intervention has had a positive effect but in a different population [37]. Additionally, their definitions state that feasibility studies have eight major focus areas: acceptability, demand, implementation, practicality, adaptation, integration, limited efficacy, and expansion. Our results indicate that LifeLab‐Soweto is acceptable to AYA, that the experience is in demand by AYA in Soweto, that implementation within a youth‐focused development centre is practical with limited resources, and that LifeLab‐UK can be adapted to a new context and expanded to cover new content. Furthermore, limited efficacy testing suggests that LifeLab‐Soweto can positively impact knowledge about BP and support the intention to positively change health behaviours. The degree to which this can become integrated into a formal education structure requires further evaluation.

## 5 Conclusion

LifeLab‐Soweto presents a promising approach to increase health literacy and to prompt review of health behaviours among adolescents and young adults in an urban South African, low‐resource setting when delivered as a short, self‐guided learning intervention. Future research should evaluate the impact of LifeLab‐Soweto in youth development centres in other parts of South Africa and determine whether integrating LifeLab‐Soweto into the school curriculum for adolescents is feasible to reinforce the learning over a longer period in a classroom environment.

## Author Contributions

**Lisa J Ware:** conceptualisation, investigation, funding acquisition, visualisation, data curation, supervision, resources, writing–original draft, writing–review and editing, validation. **Delisile Kubheka:** investigation, writing–review and editing, formal analysis, project administration. **Thato Mdladlamba:** writing–review and editing, formal analysis. **Khuthala Mabetha:** writing–original draft, writing–review and editing, formal analysis, software, validation. **Mark Hanson:** conceptualisation, writing–review and editing, methodology. **Keith M. Godfrey:** conceptualisation, methodology, writing–review and editing. **Kathryn Woods‐Townsend:** conceptualisation, methodology, writing–review and editing. **Shane Norris:** conceptualisation, funding acquisition, writing–review and editing, methodology, supervision.

## Consent

The authors have read and approved the final manuscript and consent to publication.

## Conflicts of Interest

The authors declare no conflicts of interest.

## Supporting information

Supplementary information.

Supplementary information.

## Data Availability

The data that support the findings of this study are not openly available due to reasons of sensitivity and are available from the corresponding author upon reasonable request.
