# Supplementary material for: Feasibility Testing of a Health Literacy Intervention With Adolescents and Young Adults in South Africa: The LifeLab Soweto Programme
Source: Health Expect. 2024 Dec 11;27(6):e70121. doi: 10.1111/hex.70121 (PMC11634816; doi:10.1111/hex.70121)
Supplement: Supplementary file 1 — Supplementary information. [file HEX-27-e70121-s001.docx]

Supplementary Table 1: Coding frame and examples

| **Coding Scheme** | **Examples from transcripts** |
| --- | --- |
| **Expectations about LifeLab**: Includes how engaging youth thought the program would be, what the content or format might be like, and perceptions influenced by prior experiences with healthcare services. | *Overall expectations* |
|  | I thought it would be rubbish, but I was actually interested. |
|  | I had thought it would be boring yoh, serious I thought it would be boring maybe we would sit and talk |
|  | I was expecting like more talking and no fun, but it was really fun |
|  | I didn't expect it to be so relaxing and... Fun. I thought it would be something serious. |
|  | I could tell it is a program that deals with the youth, that is what made me interested. |
|  | It was super short, and I thought it was going to be long. I came here early and I thought it was going to be long |
|  |  |
|  | *Program format expectations* |
|  | I thought maybe we'll be sitting and it will be like one on one. Educating us on health issues and what not. |
|  | At first I just thought that maybe we are going to be like fed with a bunch of information |
|  | ...my thoughts were like I would attend and then they would educate us and explain some concepts and some issues then they ask us more questions and then we get a chance to answer them |
|  | I expected that maybe we will sit and they will teach us |
|  | I expected like a boring thing like we learn, just learning. You know how.. how do I put it, the typical class. |
|  | I thought it was going to be a stressful, […] I expected us to write a test or something like or maybe they were going to check your knowledge and all that |
|  |  |
|  | *Healthcare influenced perceptions* |
|  | I thought maybe I was going to get injected to check my blood and stuff but no […] I was expecting the worst |
|  | I thought that uhm, it has something to do with doctors and stuff, yeah. So that I can get more knowledge about clinic stuff |
|  | I thought about maybe they were going to examine us check up our brains or something |
|  | I was scared ….and thought maybe you guys were going to inject us |
|  | I was thinking you guys were going to check my blood pressure and probably take my blood and check if I’m ok yah only |
|  | I expected to checked things like aids and stuff you see [… ] I then came down after seeing that those things were not there |
| **Experience of LifeLab:** Includes how youth experienced the program (including interaction with the study team), what they liked and disliked or found challenging. | *Positive feedback on program components* |
|  | ...the wire buzzer, actually like the thing is that you get to forget about other stuff, you focus there like it must not touch, like I was so focused |
|  | ...they were helpful like that one of tower [of Hanoi] I think it helped me because it had helped me boost my brain […] I think I liked the most because it gave me challenges |
|  | ...the memory game [was useful] yah because we stress a lot as teenagers, so the memory game was quite a good game for me |
|  | ...when we were reading these pamphlets, where we had to learn about the character Sifiso and the stress levels things like that, that’s what I enjoyed |
|  | The one with the blocks, what is it called?[…] Yeah tower of Hanoi […] It was helpful it relaxes your mind… |
|  | ...they are three [I really liked], the memory, wire buzzer and tower of Hanoi because those three games they involve the mind, they involve patience, they involve a lot of things in a person. |
|  |  |
|  | *Challenging program elements and negative feedback* |
|  | ...the wire buzzer was hard, it kept on going off, it was like I didn’t know what I was doing |
|  | From all the activities, it [wire buzzer] is one I didn’t like it because I failed many times while I was trying to do it |
|  | ...didn’t like? I’d say the wire buzzer […] I sucked at it, so many times |
|  | Its the one [I didn't like] where we were checking weight because I didn't want to know how much I weigh |
|  | ...there are a few instructions that I struggled with the one that measures my body waist and whereby I had to take the tape and locate my rib bone and my hip bone yes I kind of struggled there but it was not tough |
|  | When I was measuring my waist, that’s the only one I would say I didn’t read well or follow instructions, but overall I didn’t have any problem. |
|  |  |
|  | *Feedback on study team* |
|  | [With the study team] I was very comfortable because it was like I was talking to a friend |
|  | ...even when we asked and where we didn’t understand they were there to help us and make us understand. That shows that they know what they were doing |
|  | ...they could assist when I encountered problems or when I had questions, so they were there for me |
|  | ...they were really welcoming and uhm also the way they articulated that information made sense to me, it was easy to engage |
|  | ...they were so friendly, I almost felt like I was home and … what they offered I really loved […] they answered very well. There was no confusion |
|  | [The team] were good actually, they made us want to look into the programme, they made us want to know what are they actually talking about […] they wouldn't give us answers, they just explained how to do it and showed us how to improvise to get the answer |
|  |  |
|  | *Ease of use* |
|  | ...the instructions were very clear and straight forward, it was very easy to understand what was going on, yeah if you could read and understand, you could do everything |
|  | I thought that it was going to be hard or it's going to be a bit difficult but some of this things just needed you to think |
|  | ...the language was easy to understand in which they wrote. The intructions were straight to the point. You just have to read. Do whatever and yeah.. It was that easy. |
|  | I think it was easy since it actually helped by some of the people that there were there they showed you the different steps that you need to do and they were actually helpful. |
|  | It was easy for me to understand cause it talks about our, our life you see. It’s relatable, everything that we talking about there, the book. |
|  | ...everything went well […] there was some parts that were difficult but then I just had to be patient, there was some parts that were very difficult but as you read it becomes easier |
|  |  |
|  | *Overall experience* |
|  | ...my day was very fantastic, it was amazing i've learned a lot |
|  | ...it is nice here because the things done here are important, it’s not painful stuff |
|  | ...made me laugh I never expected those results |
|  | ...it made me be surprised because I did not know that such things happen. |
|  | ...it was actually fun […] I liked that it is not only about you having fun, but you actually get to learn a lot of things |
|  | I enjoyed it […] I felt like it was well equipped and well prepared I knew that I was in safe hands |
| **Learning from LifeLab**: Includes new knowledge or skills gained or a better understanding of health and/or factors that influence health, and how LifeLab supported this learning including comparison with school health education. | *New information learned - stress* |
|  | I have done a few [stress] interventions, and I thought I understand it well. [...] I know it affects your health; I didn’t think it goes that deep. |
|  | I think from that small book, that stress is not right for your health, from that character. I think some of the things I also do, I also don’t sleep well so I would say that book was helpful to me |
|  | I’ve learnt much on the stress part, […] I got much more insight about on how to deal with stress and how to identify stress. |
|  | The fact that I got to know the impact that stress has on your life. I was very unaware |
|  | ... I knew about the existence of stress but I did not know that there are different types you see, such as acute stress… All that, I did not know. |
|  | I learned things I didn't know today like stress can actually affect the future of your children and I didn't know that. |
|  |  |
|  | *New information learned - body size* |
|  | I learned that you could think you are an ideal weight, but you actually underweight and like that’s a concern for real. |
|  | I learned a lot [...] like i didn't know anything about my height, being underweight or overweight |
|  | ...today I learnt that I got an ideal weight I am not overweight yeah so it's ecstatic |
|  | Yeah I learnt that today. Things like height, people always say I'm short but then okay. Today I saw that I'm not that short |
|  | I didn’t know my height, I didn’t know my weight […] I’ve learned about myself and my body |
|  | I learnt that I should play basketball more because I am very tall |
|  |  |
|  | *New information learned - blood pressure* |
|  | I also didn't know about my BP, today I also found out about it. It made me feel good because going to the clinic sometimes yoooh it gives me nerves, I never go to the clinic |
|  | ...now I know how my blood pressure should look like because I didn’t know about it |
|  | ...now I know, I’ve got an idea of what a normal and abnormal blood pressure looks like, it’s the best one for me |
|  | ...blood pressure for me that was the most interesting part because the blood pressure thing can actually tell you more about your body and if you stressed or not and [...] smoking, that can actually affect your blood pressure |
|  | My blood pressure, because diabetes lies in my family so I'm glad I'm far from that |
|  | ...high blood pressure can affect small children as well |
|  |  |
|  | *New information learned - Concentration/focus* |
|  | I cannot concentrate very well, I noticed that it’s the only thing that is giving me problems |
|  | ...when I learned about the causes of low levels of concentration, I learned that I don’t give myself enough sleep, I have to train my mind and also exercise |
|  | I’m diagnosed with ADD so it’s not very easy for me to know and memorize things, for me to forget is simple, my concentration span is low. [...] being aware of this was going to help |
|  | I do have to sleep so that I have a clear head and be able to concentrate on my studies |
|  | I’d say it (wire buzzer) showed me a few things …that I need to improve my concentration and check myself, a lot of things I wasn’t aware of. |
|  | ...it taught me that I should be patient... I wasn't patient. […] I must be patient with the things I do more |
|  |  |
|  | *New information learned - general/other* |
|  | I’ve never been educated, you know about my health issues or exercise or you know the....epigenetics I haven’t learned about that, so yah [it was educational] |
|  | I got to learn a few things from it […] how to take care of your health […] to avoid being easily affected by diseases like high blood pressure, obesity and so on. |
|  | I learned a lot. […] I learned what they mean when they talk about health, it is not just about diseases |
|  | I was learning more and more about myself actually while reading the booklet. […] Today I saw I'm very lazy. |
|  | When I did the sit and reach I noticed I had to do something about my flexibility. |
|  | I didn't know what epigenetics is. It was very interesting. […] those we can maintain by being healthy and eating healthy, exercising, less smoking and drinking. |
|  |  |
|  | *New skills gained* |
|  | ...you allowed us to test ourselves that is what I really liked, [...] blood pressure, strength and things like that. Mostly people go to doctors now I can do that by myself |
|  | ...taking my BP all by myself. I get to be professional for once. And I was like yay! |
|  | I also like that one where we had to check our own blood pressure, height, weight because I also wish to become a nurse one day, so at least I took some information for myself |
|  | I also didn’t know how to do it [BP], it was my first time doing it today, I have never done it before in my life |
|  | ...how to control stress […] I learned that, how to take care of yourself basically, how to manage stress on a daily daily. |
|  | I think it’s, it’s good because I’ve learnt how to treat it [depression] now you see and how to exercise, yeah maybe and eat good food |
|  |  |
|  | *Comparison with school health education* |
|  | ...it wasn’t really difficult because some things weren’t new to me because I did life science at school, so it was easy to me |
|  | I did life science but ah……these things of blood pressure, I never took much interest in |
|  | I knew about genetics, yeah, I knew about some causes of stress because they teach us in school but I didn’t know about the acute stress, yeah I didn’t know that existed |
|  | It was just like the regular information we get from school, this is not good for you, you got like chronic stress, you have minor stress and such but I didn’t know like how much impact it has on you |
|  | ...stuff like epigenetics I did in school, so I also knew […] things like double helix that they unwind as genes become contagious from generation to generation, yah |
|  | I feel that teachers do not go deep to the topic, it is just for you to write and continue, but this actually helped me find out that you can actually also check yourself, like your blood pressure […]. That is things we did not do at school. |
| **Impact of LifeLab:** Includes any voiced intentions to use this learning or to change health behaviours, or altered perceptions or attitudes toward health. | *Sharing the learning* |
|  | I feel like I can teach people out there about health now. |
|  | I’m thinking of taking a new career of being a doctor coz yah it’s fun up there |
|  | I’m going to teach my family and tell them more on how to control their life and habits |
|  | I am going to encourage my friends to check out that is how I will use my knowledge and skills. I am also going to tell them the benefits, I learned more about myself than I would if I was just at home studying |
|  | ...now I can help someone if you want to check his blood pressures and measurements |
|  | I am also going to tell my friends to come here so that they can also learn more about their health |
|  |  |
|  | *Action plans for health behaviour change* |
|  | ...get enough sleep and stop sleeping those 5 hours I used to sleep and start sleeping 8 hours and more |
|  | I plan to have a reminder that will always remind me from 6am to start with push ups […] because I tend to forget. |
|  | ...this time around I’m actually going to do something, I think I will start jogging. I will be going around here […] three days a week, I can do it |
|  | ...my current weight is not right my sister, I saw that I’m overweight, I want to change it, maybe by starting gym […] I think I will join sports here at the safe hub, in the morning maybe play soccer |
|  | ...exercise, I wrote there two hours more exercise, so plus two hours of exercise […] I would change that because I know I can achieve it |
|  | ...during the weekend I just need to do morning exercises and stretch. I’m starting with yoga tomorrow |
|  |  |
|  | *Reviewing health habits* |
|  | ...now I want to take care of myself and my health. […] seeing all this information about how I impact my life I need to eat healthier than before. |
|  | I think I’m doing pretty well, but I think I need to eat more veggies |
|  | I think I have to start changing my eating habits, maybe start exercising more because I never eat a lot…...I eat and sleep and then watch TV |
|  | I’m really not happy with the fact that I smoke, I did stop a couple of time and then the boredom kicks in |
|  | I’ve noticed that stress can like affect a lot in the human body and also brain, yeah so, I think exercise is a way of taking the stress out |
|  | ...we were given the scenario of Sifiso right. I could choose to live life like Sifiso or live life differently, I guess the thing taught me that. [...] I would choose to live life differently. |
|  |  |
|  | *Altered perspectives* |
|  | I’m glad it happened because now I feel like it changes my whole view on certain things especially on the topics that we touched. |
|  | ...there are changes especially with that epigenetics part, because its not only affecting me it will go and affect my future children and grandchildren if I decide to have |
|  | I found checking your weight and waist interesting so I think I will look into being a designer |
|  | I think now I should do these games more often |
|  | ...this experience made me realize it's important to go to the clinic and check the status of your body |
|  | I never knew about going to the clinic and getting it [BP] checked or knowing your BMI and those things yes, but what I learnt today is that it is important and I should go to the clinic to check them regularly. |
