# Supplementary figures and images for: Feasibility Testing of a Health Literacy Intervention With Adolescents and Young Adults in South Africa: The LifeLab Soweto Programme
Source: Health Expect. 2024 Dec 11;27(6):e70121. doi: 10.1111/hex.70121 (PMC11634816; doi:10.1111/hex.70121)

**Supplementary Figure 1. Excerpt from the LifeLab-Soweto health literacy booklet.**


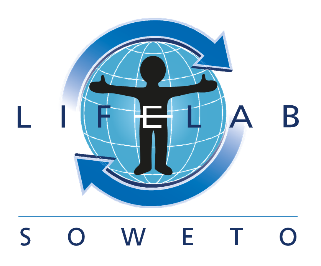


**
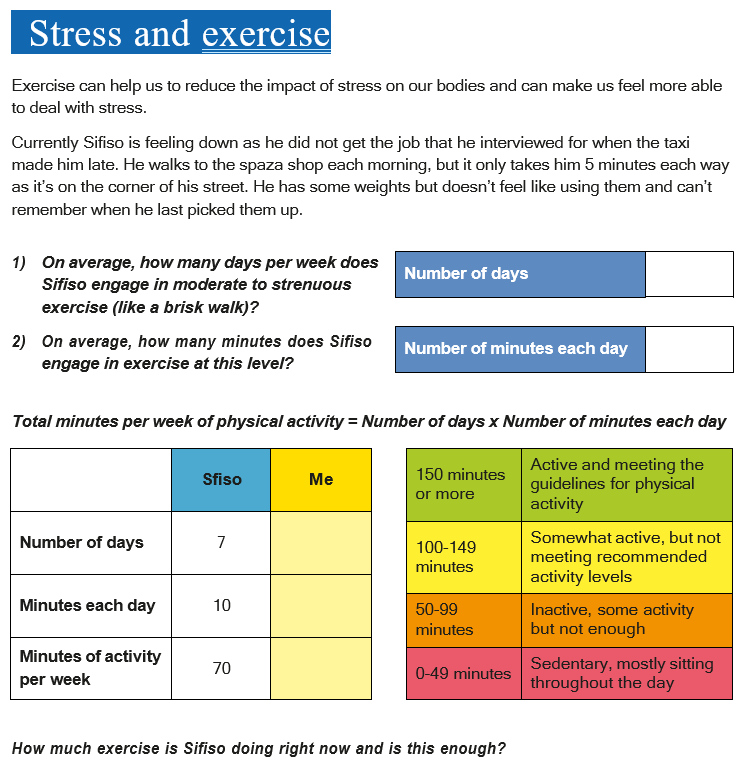
**

Supplement: Supplementary file 2 — Supplementary information. [file HEX-27-e70121-s002.docx]
